# New connections of medication use and polypharmacy with the gut microbiota composition and functional potential in a large population

**DOI:** 10.1038/s41598-024-71571-4

**Published:** 2024-10-10

**Authors:** Anna Larsson, Ulrika Ericson, Daniel Jönsson, Mariam Miari, Paschalis Athanasiadis, Gabriel Baldanzi, Louise Brunkwall, Sophie Hellstrand, Björn Klinge, Olle Melander, Peter M. Nilsson, Tove Fall, Marlena Maziarz, Marju Orho-Melander

**Affiliations:** 1 Department of Clinical Sciences in Malmö, Lund University Diabetes Center, Lund University, Malmö, Sweden; 2 Public Dental Service of Skåne, Lund, Sweden; 3 Department of Periodontology, Faculty of Odontology, Malmö University, Malmö, Sweden; 4 Molecular Epidemiology, Department of Medical Sciences, Uppsala University, Uppsala, Sweden; 5 Division of Oral Health and Periodontology, Department of Dental Medicine, Karolinska Institutet, Solna, Sweden; 6 SciLifeLab, Uppsala University, Uppsala, Sweden; 7 Clinical Research Center, Diabetes and Cardiovascular Disease, Box 50332, 202 13 Malmö, Sweden

**Keywords:** Gut microbiota, Gut metabolic modules, Medications, Polypharmacy, Population cohort, Shotgun metagenomics, Computational biology and bioinformatics, Microbiology, Medical research, Risk factors

## Abstract

Medication can affect the gut microbiota composition and function. The aim of this study was to investigate connections between use of common non-antibiotic medicines and the gut microbiota composition and function in a large Swedish cohort (N = 2223). Use of 67 medications and polypharmacy (≥ 5 medications), based on self-reported and prescription registry data, were associated with the relative abundance of 881 gut metagenomic species (> 5% prevalence) and 103 gut metabolic modules (GMMs). Altogether, 97 associations of 26 medications with 40 species and of four medications with five GMMs were observed (false discovery rate < 5%). Several earlier findings were replicated like the positive associations of proton pump inhibitors (PPIs) with numerous oral species, and those of metformin with *Escherichia* species and with lactate consumption I and arginine degradation II. Several new associations were observed between, among others, use of antidepressants, beta-blockers, nonsteroidal anti-inflammatory drugs and calcium channel blockers, and specific species. Polypharmacy was positively associated with *Enterococcus faecalis, Bacteroides uniformis, Rothia mucilaginosa*, *Escherichia coli* and *Limosilactobacillus vaginalis,* and with 13 GMMs. We confirmed several previous findings and identified numerous new associations between use of medications/polypharmacy and the gut microbiota composition and functional potential. Further studies are needed to confirm the new findings.

## Introduction

The growing understanding of the role of the gut microbiota in health and diseases like inflammatory bowel disease, neurological and autoimmune disorders, type 2 diabetes, and cardiovascular disease (CVD) has pinpointed novel paths and opportunities to improve human health through favorable modification of the gut microbiota composition and function. Although the gut microbiota composition is relatively stable in healthy adults^1^ it encompasses vast interindividual variation^2^ and can be affected by numerous environmental factors such as diet and medication ^3–5^. Some of the effects and side effects of medications may be mediated via gut bacteria and their metabolite products. Gut microbiota and microbial metabolites can also modulate the breakdown of medications and affect the response to medication or its bioavailability by influencing the human immune system or metabolic processes^6^. Polypharmacy, commonly defined as concomitant usage of five medications or more^7,8^, is increasing in parallel with an ageing population and stands for a growing cause of concern due to observed connections to a range of negative health consequences, hospitalization, and mortality^9^. Forslund et al.^10^ highlighted the importance of considering the potential confounding influence of polypharmacy to uncover true associations between disease outcomes and the gut microbiota.

Earlier, a few larger cross-sectional cohort studies, the Belgian Flemish Gut Flora (n = 1106), the Dutch Lifelines Deep (n = 1179) and the UK Biobank (n = 2737), have reported associations between self-reported medication and gut microbiota, based on 16S rRNA sequencing^3,4,11^. These studies highlighted the importance of medications as potential confounding factors in studies of the relation of gut microbiota with disease and provided evidence for association between compositional differences in the gut bacterial genera in those reporting use of medications as proton pump inhibitors (PPIs), metformin, and laxatives. More recently, a meta-analysis of three Dutch cohorts based on shotgun metagenomic sequencing, reported cross-sectional associations between 17 of the 41 included medications and gut metagenomic features, with PPIs, metformin, laxatives, and antibiotics showing the strongest associations after accounting for use of polypharmacy^12^. In line with these results, a recent Japanese study, also using shotgun metagenomic sequencing, reported associations between polypharmacy, PPIs and laxatives and the gut microbiota^5^, and further strong support has been provided by several other studies for medication-microbiome interactions for metformin^13–15^ and PPIs^16,17^.

Large general population-based cohort studies with deeply sequenced gut microbiome and high-quality medication data are scarce. Herein, we address the question of whether there are connections between use of common non-antibiotic medications and gut microbiota richness/diversity, composition and functional capacity in the population-based Malmö Offspring Study (MOS) including 2223 participants. We utilize both self-reported medication usage and nation-wide medication prescription registry data and investigate connections of medication-classes, specific medication substances and polypharmacy with the gut microbiota.

## Methods

### Description of the population cohort

MOS is a family-based population study conducted during 2013–2021 in Malmö^18^, Sweden, where adult children and grandchildren to the participants of the earlier Malmö Diet and Cancer Study Cardiovascular Cohort (MDCS-CC) were invited. No exclusion criteria were applied except difficulties in understanding information in Swedish.

Participants visited the research clinic twice. The first visit included physical examination, blood sampling (after an overnight fast) and instructions on how to collect the fecal samples at home. A web-based questionnaire on medical history of earlier disease diagnoses, ongoing medication, and lifestyle questions including leisure time physical activity level and alcohol intake was completed at home as well as a web-based 4-day food record, Riksmaten2010, designed by the Swedish National Food Agency^19^. This study includes 2644 participants with baseline data collected during the first part of MOS, March 2013–May 2017, with participation rate of 47%. Of these, 89% provided fecal samples and 68% recorded dietary intake. In total, 2223 participants with successfully performed metagenomic analyzes were included (Supplementary Fig. 1). Details about MOS have been described before^18^.

### Medication use

Information on medication use was obtained from two sources. The first was the Swedish National Prescribed Drug Register. Due to the original design of MOS, with the general aim to map factors of importance for family traits of chronic disease (e.g. CVD, diabetes, cancer, chronic obstructive pulmonary disease, and dementia), only selected medication groups had previously been requested for and approved from the National Prescribed Drug Register. These medication groups included Anatomical Therapeutic Chemical codes (ATC-codes) starting with A, C, G, H, J01, L, M, N04, N06D and R. For these, information was acquired on medication dispensation at pharmacies, during the last 12 months preceding the completion of the questionnaire, with available data for all study participants. Second, data was extracted from a questionnaire, where participants had self-reported use of all medicines during the latest week, including both prescription-based and over-the-counter medicines. Participants were additionally asked about any use of antibiotics in the past 6 months. In total, 88% of the 2223 participants answered the questionnaire.

**Fig. 1:**
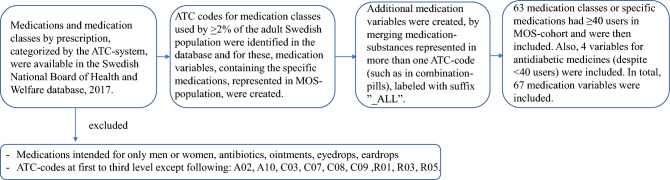
The process for selecting the medication variables for the study. From the Swedish National Board of Health and Welfare's Statistics Database for medicines, statistics on user numbers in the Swedish population were obtained, per medication and medication-class (i.e., on different ATC-levels). Medications or medication-classes used by at least 2% of the Swedish population were the base of the created medication-variables in MOS. Then, all medication-variables with ≥ 40 users in MOS (approximately 2% of the study population) were included in further analyses. Antidiabetics and metformin were added as metformin use has consistently been associated with the gut microbiota composition. Data on use of antibiotic-medications was applied to exclude individuals in sensitivity analyses.

Classification of the medications was done according to the ATC-system and the medication-variables for this study were selected in a stepwise procedure resulting in 67 non-antibiotic medication-variables as shown in Fig. 1. Participants were considered users of a medication/medication-class if this was reported in the questionnaire and/or in the prescription medication register and then coded “1” and otherwise coded “0”. The participants were categorized into three groups based on the self-reported number of non-antibiotic medications during the latest week: users of 0 medications, 1–4 medications or ≥ 5 medications, of which the latter was defined as polypharmacy.

### Metagenomic analysis of the gut microbiota

Metagenomic analysis from fecal samples was performed using a protocol that has previously described in detail^20^. In summary, fecal samples were collected by the participants at home, in sterile tubes (Sarstedt, Numbrecht, Germany) and kept in home-freezer at − 20 °C until transport to the clinic after which they were kept at − 80 °C until randomized at box-level (16 samples per box) and shipped to Clinical Microbiomics A/S (Copenhagen, Denmark), where all sample and data processing from DNA-extraction to relative abundance calculation were performed with standardized methods. In brief, DNA was extracted using NucleoSpin 96 Soil (Macherey–Nagel, Germany) including negative and positive controls (Zymogen mock) per batch during the whole laboratory process. DNA extraction quality and quantity were evaluated, and high-quality genomic DNA was randomly sheared into approximately 350 bp fragments for library construction with NEBNext Ultra Library Prep Kit for Illumina (New England Biolabs). Before sequencing, the prepared DNA libraries were purified, the fragment size distributions were evaluated, and the concentration of the final libraries were determined by quantitative real-time PCR. Metagenomic sequencing was performed with an Illumina Novaseq 6000 instrument using 2 × 150 bp paired end reads, which generated on average 26.3 million read pairs (7.9 Gb) per sample in MOS. After removal of sequencing reads with adapters, those with > 50% bases with Phred quality score < 5, and reads mapped to the human reference genome GRCh38, the remaining reads were assembled using MEGAHIT v1.1.151 and mapped using BWA mem v0.7.16a52 to a gene catalog of > 14 million non-redundant microbial genes (version “HG3A”) as earlier described^20^. Metagenomic species (MGS) were defined by the concept of binning of co-abundant genes as previously described^21^. Using this approach altogether 1985 MGS were identified of which 881, present in at least 5% of the participants, were included in the analyses. The MGS were taxonomically classified, and the resolution was mostly on species-level. The MGS count table was normalized according to the effective gene length and then normalized to 100%, resulting in estimates of relative abundance for each MGS, henceforth referred to as “species”.

The functional potential of the gut microbiota was determined by allocating genes to gut metabolic modules (GMM), including 103 metabolic pathways that represent cellular enzymatic processes^22^. Using the Omixer-RPM version 0.3.3 R package, GMM abundances were estimated, with a minimum module coverage threshold set at 66.6%.

### Oral microbiota

Of the MOS subjects, 430 additionally attended the dental arm of the Malmö Offspring Dental Study (MODS)^18^ and provided saliva samples that were used for oral microbiota analysis using the same metagenomic pipeline as for MOS fecal samples at Clinical Microbiomics A/S (Copenhagen, Denmark).

### Statistical analyses

Gut microbiota diversity was assessed using richness (number of species) the Shannon index and the inverse Simpson index. Both the Shannon and inverse Simpson indices evaluate species richness and evenness, with the Shannon index placing greater emphasis on species richness, while the inverse Simpson index focusing more on species relative abundance. These calculations were performed on the relative abundance table of all 1985 species using the *diversity* function in the vegan package in R^23^.

The overall gut microbiota composition (beta-diversity) was calculated by computing the Bray–Curtis dissimilarity index using the filtered (881 species) and rarefied data. The differences in beta-diversity between the groups were assessed using a permutational multivariate analysis of variance (using the *adonis* function in the *vegan* package with 999 permutations). We used classical multidimensional scaling (*cmdscale* in the *stats* package) to visualize the data, showing the two principal coordinates in two-dimensional space.

To investigate whether use of medications, medication-classes or polypharmacy, was associated with species or GMM relative abundances, pairwise partial Spearman’s rank correlations (*ppcor* R package) were calculated between medication-variables and species, and GMMs, adjusting for age and sex (Model 1); age, sex and body mass index (BMI) (Model 2); and age, sex, BMI, and Shannon index in the main model (Model 3). The choice of the partial Spearman correlation method was preceded by our consideration of other methods, which were deemed less suitable for following reasons: First, we assumed the medication use to affect the gut microbiota rather than the gut microbiota to have an effect on the use of medication, thus the microbiome data was the obvious outcome in the analysis. This assumption eliminated the potential use of logistic regression with medication use as the outcome. Second, we tested linear regression models with the species relative abundance as the outcome, but observed problems with spurious false positive associations, particularly with less prevalent species. Moreover, the assumption in linear regression that the outcome variable should follow a normal distribution was challenging to meet, as most species/GMMs exhibited complex distributions that could not be adequately transformed using standard methods without compromising the interpretability of the data. Shannon index was included as a covariate in the main model to reduce the risk of potentially false positive findings, since an absolute increase in one species associated with medication could produce associations of opposite direction in the relative abundance of another species and then also affect the Shannon index. We additionally adjusted for leisure time physical activity level (hereafter denoted “physical activity”) and fiber intake expressed as energy percentage (E%) (Model 4), which due to missing data in these variables reduced the sample size to 1475.

To investigate whether the alpha diversity was correlated with the use of medications or medication-classes, partial Spearman’s rank correlations were calculated adjusting for the same covariates as above (Model 1, 2) and within the reduced sample size further adjusting for physical activity and fiber intake (Model 5). The participants were divided into three categories based on the number of medications taken (0; 1–4; ≥ 5 medications). Kruskal–Wallis test was used to test whether any differences in Shannon index between the categories were statistically significant.

Three sets of sensitivity analyses were performed: excluding those using antibiotics in the last 6 months, in the last 12 months, and those using 5 or more medications. First, we excluded 262 subjects who self-reported antibiotic usage during the last 6 months as well as 364 subjects with missing self-reported antibiotic data. Secondly, we excluded 417 individuals with antibiotic use during the last 12 months, based on the combination of medication register data and self-reported antibiotic-usage the latest week. Thirdly, we excluded 76 individuals who reported use of ≥ 5 medications the latest week (polypharmacy) and 258 with missing questionnaire data on medication use.

To adjust for multiple testing, we calculated the q-values and used the Benjamini–Hochberg procedure to control the false discovery rate (FDR) at 5%. FDR was applied to each model separately, including the sensitivity analyses.

Many of the gut species associated with PPI use were species normally found in the oral cavity. For 10 of the PPI-associated gut species, corresponding species were also present from oral samples among the 430 individuals that participated both in MOS and MODS. In a post hoc analysis pairwise Spearman correlations were calculated between the relative abundances of these gut and oral species, and statistical significance was defined using FDR at 5%.

The correlation between the use of different medications was assessed using pairwise Spearman correlations.

Statistical analyses were performed in R v4.3.2 (r-project.org) and SPSS (IBM SPSS Statistics 28.0) and visualized in R (using ggplot2)

## Results

### Clinical characteristics of the Malmö Offspring Study cohort and medication use

The age range of the 2223 included participants was 18–70 years, 52% were females and 42% reported use of at least one medication the latest week (Table 1). The most frequent medications in MOS were nonsteroidal anti-inflammatory drugs (NSAIDs) (M01A), used by 17% of the participants (Table 2). Co-occurrence of medication use is presented in Supplementary Fig. 2.

**Table 1:** Characteristics of participants in the Malmö Offspring Study.

Variables	N	All^a^ N = 2223	Self-reported drug usage, latest week^b^			*P-*value		
			No drugs N = 1026	1–4 drugs N = 863	≥ 5 drugs (polypharmacy) N = 76	No drugs vs polypharmacy	1–4 drugs vs polypharmacy	0–4 drugs vs polypharmacy
Age (years)	2223	40.4 ± 13.9	37.8 ± 13.0	43.2 ± 14.0	50.3 ± 12.7	**1.6E−12**	**1.2E−05**	**2.3E−09**
Sex (women)	2223	1159 (52.1)	487 (47.5)	513 (59.4)	47 (61.8)	**0.02**	0.77	0.16
BMI (kg/m^2^)	2223	25.9 ± 4.7	25.3 ± 4.4	26.4 ± 5.0	29.0 ± 4.8	**2.5E−9**	**1.6E−05**	**1.2E−07**
Current smoker	1958	310 (15.8)	159 (15.6)	140 (16.3)	11 (14.5)	0.93	0.81	0.86
Alcohol intake 4 times per week or more	1953	75 (3.8)	31 (3.0)	41 (4.8)	3 (3.9)	0.73	> 0.99	0.77
Leisure time physical activity level, sedentary or low	1947	906 (46.5)	454 (44.7)	403 (47.0)	49 (66.2)	**5.4E−04**	**2.2E−03**	**8.3E−04**
Education level, university	1954	731 (37.4)	373 (36.3)	333 (38.7)	25 (33.3)	0.65	0.43	0.53
Fibre intake (E%)	1607	1.87 ± 0.63	1.84 ± 0.63	1.89 ± 0.60	1.95 ± 0.62	0.26	0.53	0.36
Antibiotic use in the last 6 months, self-reported	1859	262 (14.1)	112 (11.7)	130 (15.7)	20 (27.0)	**3.0E−04**	**0.02**	**2.0E−03**
Antibiotic use in the last 12 month^c^	2223	417 (18.8)	140 (13.6)	193 (22.4)	25 (32.9)	**1.2E-05**	**0.05**	**1.2E−03**
Prevalent disease								
Diabetes	1902	53 (2.8)	3 (0.3)	34 (4.0)	16 (21.3)	**7.9E−17**	**9.2E−10**	**8.1E−22**
Hypertension	1903	348 (18.3)	84 (8.6)	217 (25.5)	47 (62.7)	**1.9E−41**	**2.1E−11**	**1.7E−23**
Myocardial infarction	1895	19 (1.0)	1 (0.1)	6 (0.7)	12 (16.2)	**8.1E−14**	**1.4E−18**	**1.9E−37**
Stroke	1896	19 (1.0)	3 (0.3)	15 (1.8)	1 (1.4)	0.25	> 0.99	0.53
Celiac disease	1893	23 (1.2)	8 (0.8)	14 (1.7)	1 (1.3)	0.49	> 0.99	0.61
IBS	1899	320 (16.9)	118 (12.1)	179 (21.0)	23 (30.7)	**1.3E−05**	0.07	**1.9E−03**
Crohn's disease	1897	13 (0.7)	2 (0.2)	9 (1.1)	2 (2.7)	**0.03**	0.22	0.09
Ulcerative colitis	1898	19 (1.0)	4 (0.4)	10 (1.2)	5 (6.7)	**1.7E−04**	**4.8E−03**	**6.4E−04**
Cancer	1897	75 (4.0)	25 (2.6)	43 (5.1)	7 (9.3)	**3.3E−03**	0.20	**0.03**

^a^Data is given as mean ± SD or N (%). *E%* energy percentage, *BMI* body mass index, *IBS* Irritable bowel syndrome. ^b^258 participants did not report. ^c^Combined self-reported- (latest week) and drug register data from the latest 12 month: users coded as 1, all others coded as 0. *P*-values comparing the drug-usage groups are from the t-test for continuous variables and from Chi-squared test for the categorical variables, except for in comparisons including cells < 5 observations they are from Fisher exact test. Significant *p*-values (< 0.05) are in bold.

**Table 2:** Frequencies of medication users in the Malmö Offspring Study (N = 2223).

Medication ATC-code	Variable name^a^	Number of users^b^(%)
**Antiinflammatory and antirheumatics non steroids** **M01A**	**NSAID_and_Glucosamine_M01A**	**379 (17.0)**
Propionic acid derivatives (NSAID) M01AE	NSAID_M01AE	233 (10.5)
Ibuprofen M01AE01	NSAID_Ibuprofen_M01AE01	173 (7.8)
Naproxen M01AE02	NSAID_Naproxen_M01AE02	46 (2.1)
Acetic acid derivatives and related substances M01AB	NSAID_M01AB	142 (6.4)
Diclofenac M01AB05	NSAID_Diclofenac_M01AB05	137 (6.2)
**Antihistamines for systemic use** **R06A**	**Antihistamines_Oral_R06A**	**238 (10.7)**
Other antihistamines R06AX	Antihistamines_Oral_Other_R06AX	168 (7.6)
Desloratadine R06AX27	Antihistamines_Oral_Desloratadine_R06AX27	95 (4.3)
Loratadine R06AX13	Antihistamines_Oral_Loratadine_R06AX13	63 (2.8)
**Drugs for acid related disorders** **A02**	**Drugs_acid_related_disorders_A02**	**225 (10.1)**
Drugs for pepctic ulcer and gastro-oesophageal reflux disease A02B	Drugs_peptic_ulcers_reflux_A02B	215 (9.7)
PPI A02BC	PPI_A02BC	201 (9.0)
Omeprazol A02BC01	PPI_Omeprazol_A02BC01	166 (7.5)
**Other analgesics and antipyretics** **N02B**	**Other_analgetics_antipyretics_N02B**	**217 (9.8)**
Paracetamol, plain N02BE01	Paracetamol_N02BE01	178 (8.0)
**Paracetamol, incl combinations** **N02AJ06, N02BE01**	**Paracetamol_ALL**	**187 (8.4)**
**Nasal preparations** **R01**	**Nasal_preparations_R01**	**183 (8.2)**
Decongestants and other nasal preparations for topical use R01A	Nasal_topical_R01A	161 (7.2)
Corticosteroids nose R01AD	Corticosteroids_Nasal_R01AD	155 (7.0)
Mometasone R01AD09	Corticosteroids_Nasal_Mometasone_R01AD09	111 (5.0)
Phenylpropanolamine R01BA01	Phenylpropanolamine_R01BA01	40 (1.8)
**Other drugs for obstructive airway diseases, inhalants** **R03**	**Drugs_obstructive_ariways_R03**	**171 (7.7)**
Adrenergics inhalants R03A	Adrenergics_Inhalants_R03A	152 (6.8)
Selective beta-2-adrenoreceptor agonists R03AC	Adrenergics_Beta2_agonist_Inhalants_R03AC	109 (4.9)
Terbutaline R03AC03	Terbutaline_inhalants_R03AC03	68 (3.1)
Adrenergics in combination with corticosteroids or other drugs, excl. Anticholinergics R03AK	Adrenergics_combinations_inhalants_R03AK	68 (3.1)
Formoterol and budesonide, combination R03AK07	Formoterol_budesonide_inhalants_R03AK07	56 (2.5)
Inhalation steroids R03BA, R03AK	Inhalation_steroids_ALL	121 (5.4)
Budesonide R03BA02	Budesonide_Inhalants_R03BA02	53 (2.4)
**Agents acting on the renin-angiotensin system** **C09**	**Agents_RAS_system_C09**	**158 (7.1)**
ARB incl combinations C09C, C09D	ARBs_ALL	91 (4.1)
ARB plain C09CA	ARBs_C09CA	77 (3.5)
Losartan C09CA01	Losartan_C09CA01	43 (1.9)
ACE-inhibitors inc. Combinations C09A, C09B	ACEi_ALL	71 (3.2)
**Cough and Cold preparations** **R05**	**Cough_Cold_preparations_R05**	**149 (6.7)**
Cough suppressants and expectorants, combinations R05F	Cough_suppress_expectorants_R05F	98 (4.4)
Opium derivatives and expectorants R05FA02	Cough_mixture_Opium_derivate_R05FA02	93 (4.2)
Expectorants, excl. Combinations with cough supressants R05C	Expectorants_excl_cough_supressants_R05C	63 (2.8)
Mucolytics_comb R05CB10	Mucolytics_comb_R05CB10	41 (1.8)
**Antidepressants** **N06A**	**Antidepressants_N06A**	**129 (5.8)**
SSRI Selective serotonin reuptake inhibitors N06AB	Antidepressants_SSRI_N06AB	92 (4.1)
Sertraline N06AB06	Sertraline_N06AB06	51 (2.3)
**Lipid modifying agents** **C10A**	**Lipid_lowerers_C10A**	**123 (5.5)**
Statins C10AA	Statins_C10AA	113 (5.1)
Simvastatin C10AA01	Simvastatin_C10AA01	66 (3.0)
Atorvastatin C10AA05	Atorvastatin_C10AA05	48 (2.2)
**Beta blocking agents** **C07**	**Betablockers_C07**	**112 (5.0)**
Beta blocking agents, selective C07AB	Beta_blockers_selective_C07AB	94 (4.2)
Metoprolol incl combinations C07AB02, C07FB02	Metoprolol_ALL	71 (3.2)
**Corticosteroids systemic** **H02A**	**Corticosteroids_oral_H02A**	**110 (4.9)**
Glucocorticoids H02AB	Glucocorticoids_oral_H02AB	109 (4.9)
Betamethasone H02AB01	Betamethasone_oral_H02AB01	61 (2.7)
**ASA, merged** **N02BA01, B01AC06,N02BA51, N02AJ09**	**ASA_ALL**	**86 (3.9)**
Platelet aggregation inhibitors excl. Heparin B01AC	**Platelet_aggregation_inhibitors_B01AC**	**43 (1.9)**
ASA_anticoag B01AC06	ASA_anticoag_B01AC06	40 (1.8)
**Levothyroxine sodium** **H03AA01**	**Levothyroxine_H03AA01**	**86 (3.9)**
**Calcium channel blockers** **C08**	**Calcium_channel_blockers_C08**	**71 (3.2)**
Amlodipine C08CA01	Amlodipine_C08CA01	44 (2.0)
**Drugs for constipation** **A06A**	**Laxatives_A06A**	**70 (3.1)**
**Drugs used in diabetes** **A10**	**Antidiabetics_A10**	**53 (2.4)**
Blood Glucose Lowering (excluding Insulines) A10B	Antidiabetics_not_Insulins_A10B	38 (1.7)
Metformin A10BA02 A10BD20	Metformin_ALL	36 (1.6)
Insulins and analouges A10A	Insulins_and_analouges_A10A	23 (1.0)
**Thiazides incl combinations** **C03A, C03EA, C09BA, C09DA**	**Thiazides_ALL**	**52 (2.3)**
**Diuretics** **C03**	**Diuretics_C03**	**42 (1.9)**

Medication class headings in bold, followed by subgroups in hierarchical ATC-order. ^a^Variable names correspond to those in Fig. 3. ^b^Combined self-reported- (latest week) and drug register data from the latest 12 months: users coded as 1, all others coded as 0.

Compared to participants without any medications or with less than five medications, those with polypharmacy (≥ 5 medications) were significantly older, were more often women, had higher BMI and lower physical activity level, and a higher prevalence of diabetes, hypertension, previous myocardial infarction, IBS, Crohn's disease, ulcerative colitis, and cancer (Table 1). Use of antibiotics was also higher among those with polypharmacy (Table 1). Altogether, 147 different medications (individual ATC-codes) were used by the 76 participants with polypharmacy, where paracetamol, acetylsalicylic acid, metoprolol, atorvastatin, and metformin were the most common medications.

### Medication use and the gut microbial diversity and overall composition

**Fig. 2:**
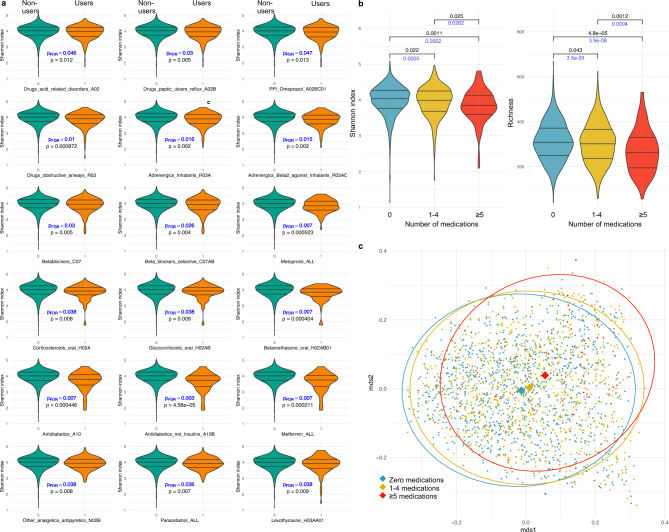
Medication use and the gut microbial richness and evenness, and overall composition. (**a**) Violine plots of Shannon index in participants stratified by users (orange) and non-users (green) of 18 medications or medication-classes with correlations at q < 0.05, adjusted for age, sex, and BMI. Unadjusted P-values (in black) and FDR adjusted q-values (in blue) refer to partial Spearman correlation analyses. The three horizontal lines indicate the median and the 25th and 75th percentiles (interquartile range) of Shannon index of users and non-users of medications or medication classes. (**b**) Shannon index and richness of participants stratified by the number of medications used; 0, 1–4 or ≥ 5 (polypharmacy). Kruskal–Wallis test was used to calculate differences in Shannon index and richness between the three groups. P-values are presented for analyses before (in black), and after adjustments for sex, age, and BMI (in blue). The three horizontal lines indicate the median and the 25th and 75th percentiles (interquartile range) of Shannon index. (**c**) The two principal coordinates (mds1 and mds2, obtained using multidimensional scaling (MDS) analysis) of the Bray–Curtis dissimilarity index (beta-diversity) for participants stratified by the number of medications used. Each dot represents one individual, and colors indicate the group. Dissimilarities in the overall microbial community between the three groups were calculated by permutation test (R^2^ = 0.002, p < 0.001).

Lower richness, Shannon index and/or inverse Simpson were observed among users of 26, 18 and 2 medications or medication-classes based on partial Spearman correlation (q < 0.05 for all) as compared to non-users adjusting for age, sex, and BMI (Supplementary Table 10), with highly overlapping results for richness and Shannon, of which the latter results are presented in Fig. 2a. All diversity measures were decreased in participants with polypharmacy as compared to individuals without adjusting for age, sex, and BMI (p = 2.71e−6, p = 0.0003 and p = 0.019, respectively, Supplementary Table 24) with group-wise comparisons presented for Shannon index and richness in Fig. 2b. Assessment of inter-individual differences in the overall gut microbiota composition by beta-diversity additionally revealed dissimilarities in the overall microbial community between the medication-use categories in a permutational multivariate analysis of variance (p = 0.001) (Fig. 2c).

### Associations between medication use and gut species

**Fig. 3:**
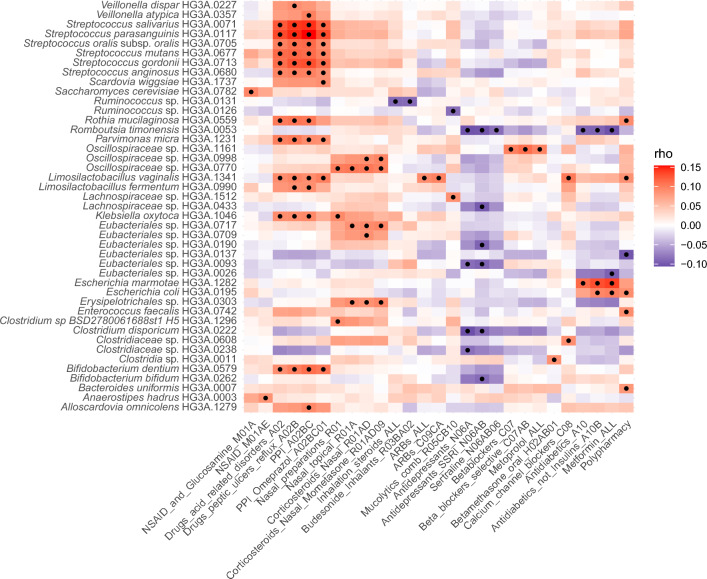
Associations between gut species and medication use. The heatmap displays partial Spearman’s rank correlations for all medications/medication-classes and polypharmacy (columns) that were significantly associated with any of the gut species (rows) adjusting for age, sex, BMI, and Shannon index. All correlations with q < 0.05 are indicated by •. Red indicates positive and blue negative correlations.

Using the partial Spearman correlations adjusted for age, sex, BMI, and Shannon index we observed 97 significant associations between 26 different medications/medication-classes and abundance of 40 individual species (q < 0.05) (Fig. 3, Supplementary Table 3), and the majority (80%) of the associations were positive. Prevalence and full taxonomy of the associated species is presented in Supplementary Table 29.

The highest number of associations (q < 0.05), altogether 48, were observed between 16 different species and use of the medication-class ‘Drugs for acid related disorders’ or its ATC-subordinates (ATC A02, A02B, A02BC or A02BC01). In medication users belonging to this group, higher relative abundance of several *Streptococcus* species was observed as were higher relative abundances of *Bifidobacterium dentium, Rothia mucilaginosa*, two *Veilonella* species, two *Limosilactobacillus* species, *Klebsiella oxytoca*, *Parvimonas micra*, *Allsocardovia omnicolens* and *Scardovia wiggsiae*. For ten of these PPI-associated gut species we identified the oral counterpart species in MODS saliva metagenomic data. The relative abundance of all these ten gut species correlated (p < 0.05) with the abundance of corresponding species from the oral microbiota (Table 3).

**Table 3:** Correlations between relative abundances of gut species that associated with drugs for acid related disorders and their corresponding oral counterparts in 430 participants of the Malmö Offspring Study (MOS) and Malmö Offspring Dental Study (MODS) participants.

Gut species	Oral species	ρ	p
Rothia mucilaginosa HG3A.0559	Rothia mucilaginosa Ho1A.0117	0.55	2.89E−35*
Rothia mucilaginosa HG3A.0559	Rothia mucilaginosa Ho1A.0001	− 0.09	7.04E−02
Scardovia wiggsiae HG3A.1737	Scardovia sp. Ho1A.0098	0.33	1.66E−12*
Alloscardovia omnicolens HG3A.1279	Alloscardovia omnicolens Ho1A.0123	0.29	1.68E−09*
Streptococcus parasanguinis HG3A.0117	Streptococcus parasanguinis Ho1A.0005	0.28	2.73E−09*
Veillonella dispar HG3A.0227	Veillonella dispar Ho1A.0018	0.27	2.06E−08*
Veillonella atypica HG3A.0357	Veillonella atypica Ho1A.0013	0.25	1.09E−07*
Bifidobacterium dentium HG3A.0579	Bifidobacterium dentium Ho1A.0146	0.24	7.19E−07*
Streptococcus gordonii HG3A.0713	Streptococcus gordonii Ho1A.0065	0.21	7.17E−06*
Streptococcus salivarius HG3A.0071	Streptococcus salivarius Ho1A.0002	0.15	2.00E−03*
Streptococcus salivarius HG3A.0071	Streptococcus salivarius Ho1A.0234	0.06	2.08E−01
Parvimonas micra HG3A.1231	Parvimonas micra Ho1A.0046	0.13	7.83E−03*

ρ is the Spearman correlation coefficient and p is the nominal p-value for the correlation. Bold indicates statistical significance at 0.05 for unadjusted p-values and * indicates statistical significance at FDR 5%.

Among the strongest associations were those with medications used in diabetes, in particular metformin. Higher relative abundance of *Escherichia marmotae* and *E. coli* and lower relative abundance of *Romboutsia timonensis* were observed in users of metformin (q < 10^–7^) and all these associations were found to be stronger at the substance level (metformin) rather than on the superior medication class levels. Similarly to users of metformin, users of antidepressants, selective serotonin reuptake inhibitors (SSRIs), and/or sertraline had a lower abundance of *R. timonensis*. Only negative associations were seen between use of antidepressants and gut bacterial species, with lower abundance of *Clostridium disporicum* (HG3A.0222) and *Eubacteriales sp*. (HG3A.0093) in users of antidepressants and SSRI, *Lachnospiraceae sp*. (HG3A.0433), *Bifidobacterium bifidum*, and *Eubacteriales sp*. (HG3A.0190) in users of SSRI, and *Clostridiaceae sp.* (HG3A.0238) in users of antidepressants.

Further associations were observed between usage of beta-blockers, selective beta-blockers and metoprolol and higher abundance of *Oscillospiraceae* sp. (HG3A.1161). In users of NSAIDs (ATC M01A), the fungus *Saccharomyces cerevisiae* was observed in higher abundance while *Anaerostipes hadrus* (HG3A.0003) was seen in higher abundance in users of propionic acid derivative types of NSAIDs.

Among users of nasal preparations, a higher abundance of seven species was detected. Nasal corticosteroids were associated with five of these species including two *Oscillospiraceae* species (HG3A.0770 and HG3A.0998), two *Eubacteriales* species (HG3A.0717 and HG3A.0709) and *Erysipelotrichales* sp. (HG3A.0303). In addition, nasal preparations were associated with higher abundance of *K. oxytoca* and *Clostridium* sp. (BSD2780061688st1.H5). Concerning oral corticosteroids, users of betamethasone were observed to have higher abundance of *Clostridia* sp. (HG3A.0011)*.* Use of any inhalation steroids and specifically budesonide inhalants associated with lower abundance of *Ruminococcus* sp*.* (HG3A.0131), while use of combined mucolytic cough mixtures associated with lower abundance of *Ruminococcus* sp*.* (HG3A.0126) and higher abundance of *Lachnospiraceae* sp*.* (HG3A.1512)*. L. vaginalis*, with a prevalence of only 5.4% (females 4.3%, males 6.6%), that was observed to be more abundant among users of PPIs, was additionally positively associated with use of angiotensin II receptor blockers (ARBs) and with calcium channel blockers, of which the latter additionally associated with a higher abundance of *Clostridiaceae* sp*.* (HG3A.0608).

Polypharmacy associated with lower abundance of *Eubacteriales* sp. (HG3A.0137) and with higher abundance *Enterococcus faecalis, Bacteroides uniformis, R. mucilaginosa*, *E. coli* and *L. vaginalis,* of which the last three also associated with use of individual medications/medication-classes as presented above (Fig. 3). In the model adjusted for age, sex, and BMI but not for Shannon index (model 2, Supplementary Table 17), 61 associations were observed (q < 0.05) between polypharmacy and species, of which 72% were negative (Supplementary Fig. 3).

### Associations between medication use and the functional potential of the gut microbiota

**Fig. 4:**
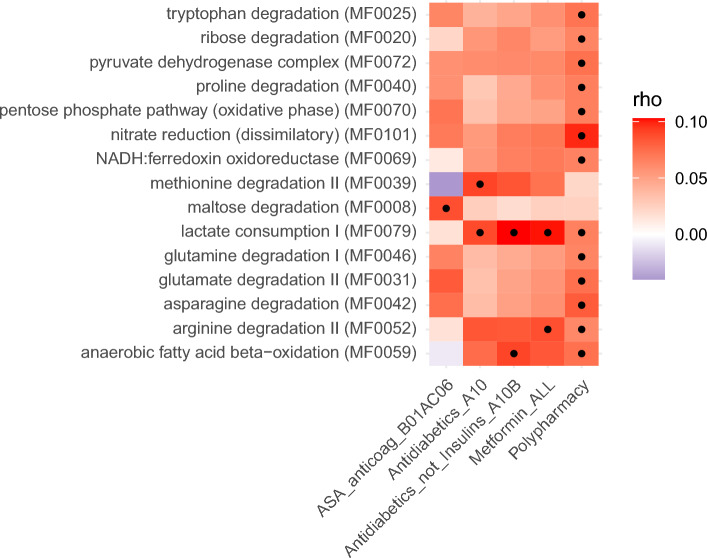
Associations between gut metabolic modules (GMMs) and medication use. The heat map displays partial Spearman’s rank correlations for all medications/medication classes and polypharmacy (columns) that were significantly associated with any of the GMMs (rows), adjusted for age, sex, BMI, and Shannon index. All correlations with q < 0.05 are indicated by • . Red indicates positive correlations, and blue indicates negative correlations.

**Fig. 5:**
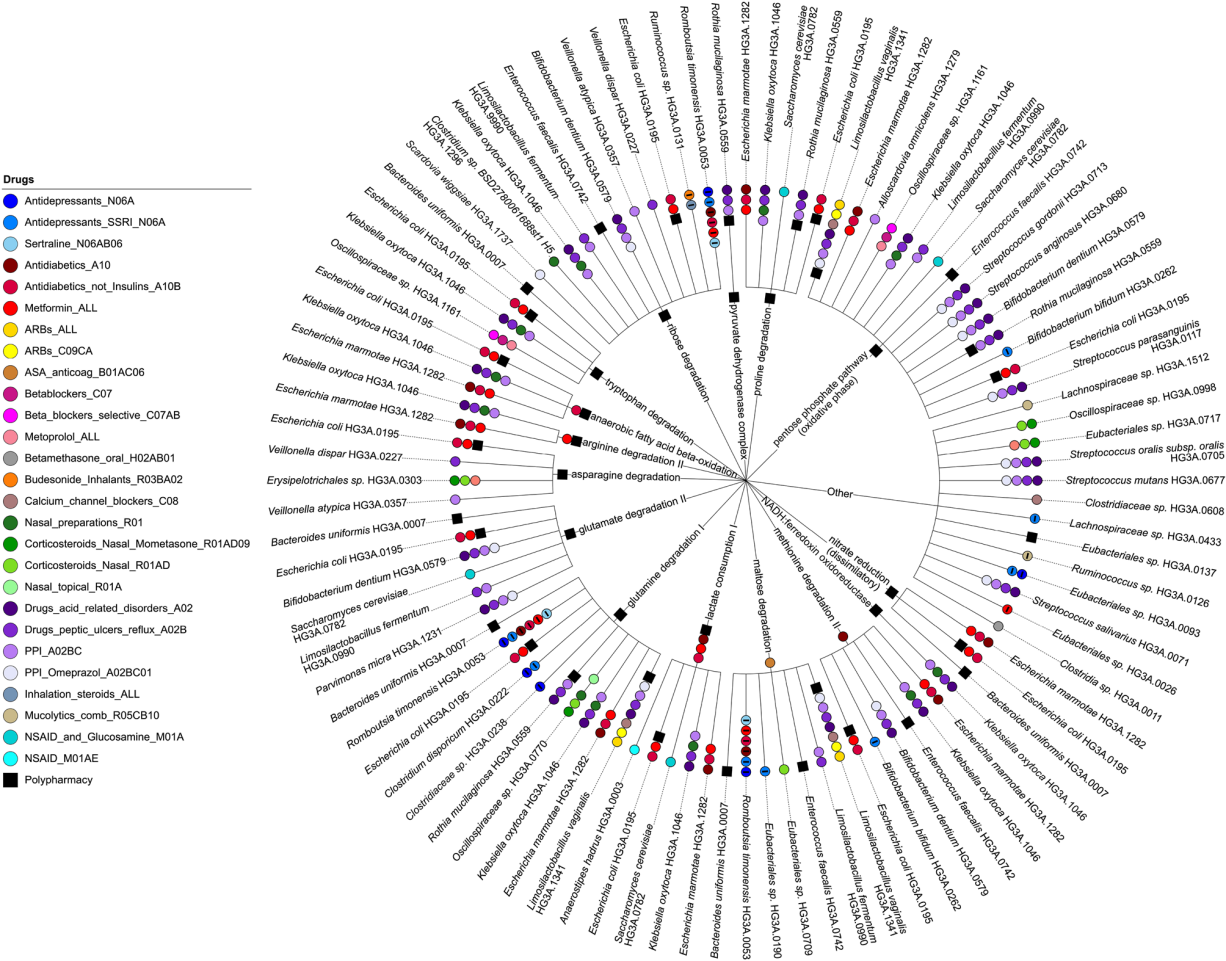
Overview of the associations between medication use and gut species and gut metabolic modules (GMMs), and their connections. The inner circle shows all GMMs that are associated with medications or polypharmacy in Fig. 4, and the outer circle shows the species that harbor these GMMs amongst those species that are associated with any medication or polypharmacy in Fig. 3. Each colored bullet on the inner or outer circle represents specific medications or polypharmacy that are associated with a GMM or a species, respectively. Bullets that contain a minus sign refer to a negative association, all the others are positive associations. The plot was produced by using iTOL^50^.

In total, we found associations between 15 GMMs, each representing unique microbial functions, and four medications or medication classes or polypharmacy, using partial Spearman correlations adjusted for age, sex, BMI and Shannon index (q < 0.05, Fig. 4, Supplementary tables 34–48). Of these, lactate consumption I and arginine degradation II were positively associated with use of metformin, and methionine degradation with anti-diabetic medication class, while maltose degradation associated with use of acetylsalicylic acid (ASA). Altogether 10 GMMs were positively associated (q < 0.05) solely with polypharmacy. As different species may harbor genes that enable them to perform the same metabolic function, we combined the results of GMM and species associations with medication or polypharmacy usage in Fig. 5, to enable easier interpretation of both results together. For example, in metformin users we observed elevated abundance of lactate consumption I and arginine degradation II and both these GMMs are harbored by *E. marmotae*, a species with higher relative abundance (q < 0.05) in metformin users (Fig. 5). Among the elevated GMMs in polypharmacy users, the strongest association (q = 0.001) was observed for dissimilatory nitrate reduction, aligning with *E. coli* and *B. uniformis* which harbor these functions and associate with polypharmacy in our study (Fig. 5). Most of the polypharmacy associated functions belong to pathways encoding amino acid degradation, or energy metabolism and the five species that positively associated with polypharmacy in our study harbored between three and 11 of the GMMs that associated with polypharmacy.

### Additional adjustments for fiber intake and leisure time physical activity level, and sensitivity analyses

These results are presented in the Supplementary information file (Supplementary results).

## Discussion

In a large Swedish population-based cohort, we report associations between specific gut species and functional GMMs and commonly used medications/medication-classes and polypharmacy. We replicate earlier associations between use of PPIs, metformin, and SSRIs with specific gut species, and describe new associations with use of metformin, antidepressants/SSRIs, NSAIDs and NSAID-subtypes, ARBs, calcium channel blockers, beta-blocking agents, nasal preparations, inhaled or oral steroids and polypharmacy. Regarding metformin, we replicate earlier findings of increased lactate consumption I and arginine degradation II in users of this drug, and present new observations of elevated methionine degradation and anaerobic fatty acid oxidation in users of antidiabetic medications. We replicate previously described negative associations between Shannon index and use of PPI, paracetamol, metformin, and metoprolol and report new negative associations for inhalants for obstructive airways, oral corticosteroids, and levothyroxine. Further, among individuals with polypharmacy, we report differences in the overall gut microbial composition and decreased richness and Shannon index, replicating previous findings^5^, and describe both new and replicated associations between polypharmacy and specific gut species, of which some did not associate with any other specific medications. We further report numerous associations with deviations in the functional potential of the gut microbiome among those with polypharmacy, most of which were solely associated with polypharmacy and not with use of any individual medication.

Earlier studies of associations between medication use and gut microbiota using shotgun metagenomics in large cohorts have been scarce^5,12,24^. MOS cohort represents the general population and both registry data on prescription medicines and self-reported medication use in the past week, including over-the-counter medications, were utilized. As not all medication-classes were available from the medication register due to the original design of MOS, the self-reported data added valuable complementary information, also regarding ongoing concomitant medication-use.

Lower gut microbiota alpha diversity, commonly measured as richness (number of species) and richness and evenness (Shannon index), has been observed in numerous diseases^11,25^ as well as among users of specific medications^5,11,17,24,26,27^. We observed lower gut microbiota richness amongst users of almost 40% of the investigated medications, and 70% of these additionally associated with lower Shannon index. In line with Jackson et al.^11^, we found lower Shannon index in users of metformin, paracetamol, and PPI. Contrary to our results, Nagata et al.^5^ observed a higher Shannon index in PPI users, and our associations did not withstand the sensitivity-analyses, which showed substantial reductions in the correlation coefficients and thus the association might rather reflect previous antibiotic use or occurrence of polypharmacy. The associations of paracetamol and metformin with Shannon index did not withstand sensitivity analysis excluding polypharmacy users, which could reflect either decreased power as these medications were amongst the most common among those with polypharmacy, or an impact of polypharmacy on Shannon index, or both. In our study, lower richness and Shannon index were also seen among users of beta-blockers, replicating earlier result in the Estonian microbiome cohort^24^. Further, we observed lower richness and Shannon index in users of oral corticosteroids, and medications for obstructive airways, and decreased Shannon index amongst users of levothyroxine, which to our knowledge have not been reported before. The strengths of these correlations were generally preserved in the three sensitivity analyses. Overall, the further adjustment for fiber intake and physical activity were not observed to have any major impact on the associations between medications or polypharmacy and Shannon index.

Our study provides further convincing evidence, put forward in several earlier studies, for a strong connection between PPI use and gut microbiota. Concordant with earlier observational studies, a higher relative abundance of species of oral origin, like those of *Streptococcus* and *Rothia* genera, was characteristic for the gut microbiota of PPI users in our study^5,12,16,17,26^. By repeated sampling, Nagata et al.^5^ demonstrated increased relative abundance of *Streptococcus* in the gut microbiota in individuals after starting with PPI, and a corresponding decrease after termination. This has been suggested to be mediated by the increased pH in the gut due to PPI use, which can promote colonization of certain bacteria at more distal parts of the digestive tract^10,26,28^.

Most gut species with elevated abundance among PPI-users are normal habitants of the human oral flora. For 10 of 16 such species in the gut, we identified oral counterparts in MODS, and the relative abundance of all these correlated with the corresponding oral species, which is in line with the view that PPI use may promote transferal of certain oral species to the gut.

Both observational and interventional studies have demonstrated specific changes in the gut microbiota of metformin users and a direct anti-diabetic effect mediated by gut bacteria has been demonstrated by fecal transplantation from metformin treated donors to germ-free mice^14^. Both improved glucose control and negative side effects of metformin have previously been attributed to gut microbiota mediated mechanisms^12,13,29^. Results from our study provide support for metformin-related changes in the gut microbiota. We observed a higher relative abundance of two *Escherichia* species, *E. marmotae* and *E. coli,* among users of metformin, of which the association with *E. coli* has repeatedly been described in previous studies^10,13,14,29^. *E. marmotae* is a recently described species, phenotypically indistinguishable from *E. coli*, that in most earlier studies likely has been misidentified as *E. coli*^30^. Consistent with previous studies, we also observed nominally decreased abundance of *I. bartlettii* in metformin users^13,14^ and support for a causal connection was recently provided by a randomized trial where initiation of metformin in treatment of naïve adults resulted in decreased abundance of *I. bartlettii*^29^. Our study additionally identified *R. timonensis* in lower abundance among metformin users. Like *E. marmotae*, *R. timonensis* is a newly isolated species from the human gut^31^, and has not been captured in earlier data-base dependent gut microbiota studies. However, this finding is supported by another recent large metagenomic study from Sweden where metformin use was based on plasma metabolome data^20^.

A major feature of the gut microbiome is the metabolic functional potential which we investigated utilizing GMMs, representing metabolic modules that are assigned based on known functions of bacterial genes^22^. Apart from the numerous GMM associations with polypharmacy, discussed later, associations with specific medications were restricted to antidiabetic medications including metformin, and ASA, of which the latter did not associate with any specific species in our study. In users of metformin, lactate consumption I was elevated, which is in line with associations of metformin with *E. coli* and *E. marmotae,* that harbor these functions, in our study. Metformin was further associated with arginine degradation II harbored by *E. marmotae.* In line with our results, Wu et al.^14^ observed that initiation of metformin treatment led to upregulation of bacterial amino acid metabolism, including arginine degradation. They further found increased fecal concentrations of lactate in the metformin-treated group, as compared to the placebo group, after 4 months of treatment. Here, we found an increase in lactate consumption I, and it is possible that the increased lactate levels in feces may benefit the expansion of species that can utilize lactate. Further evidence concordant with our results is by Nagata et al.^5^ reporting all genes related to lactate consumption I and arginine degradation II elevated in Japanese metformin users.

Gut microbiota interacts bilaterally with the central nervous system via the gut-brain axis^32^ and has been associated with depression^33,34^. Previous association studies of antidepressants and gut microbiota have provided diverging results. We solely found negative associations between antidepressants and gut species, which is in line with a recent study showing anti-microbial activity of several antidepressants in vitro on specific habitants of normal gut microbiota, including *Bifidobacterium bifidum*^35^, which we found in decreased abundance among SSRI users. All other SSRI associated species in our study belong to *Clostridia*, and two of them belong to *Clostridiaceae* family, which associated with SSRI use in an earlier study^11^.

Changes in the gut microbiota as a consequence of use of subclasses of NSAIDs have been described in numerous animal studies, while large human metagenomic population studies are lacking^36^. Of our study participants, 17% used NSAIDs and this was associated with increased abundance of *Saccharomyces cerevisiae*, a new finding that needs confirmation in other studies. *S. cerevisiae* is a common yeast in fermented food and a frequent habitant of the gut flora^37^ and has been attributed potential positive health outcomes such as protection of the gut mucosa and stimulation of the immune system^38^. Anti-saccharomyces antibodies have been described as predictors of inflammatory bowel disease progress^39^. One of two subspecies of *Anaerostipes hadrus* (HG3A.0003) was found in higher abundance among users of propionic acid derivative types of NSAIDs, while this species did not show any association with use of acetic acid derivative types of NSAIDs nor with the subordinate substance diclofenac. Previously, NSAID-type specific gut microbiota associations have been reported in a smaller study utilizing 16S sequencing^40^, while no associations with NSAID use was found in a larger metagenomic study that did not differentiate between different types of NSAIDs^12^. Further gut metagenomic studies and functional analyses are needed to elucidate the interconnectedness between the different NSAIDs, the gut microbiota and clinical outcomes.

In rodents, the antihypertensive effect of ARBs has been connected to beneficial changes in the gut^41,42^. In our study we found increased relative abundance of *L. vaginalis* in users of both ARBs and calcium channel blockers, which is in line with increase of *lactobacilli* in the gut of mice treated with these medications^41,43^, while these associations in humans are new. However, blood pressure lowering effect of probiotics containing *Lactobacillus* spp. has been shown in human studies and animal models^44^. Another new finding of our study was the robust association between use of beta-blockers and increased abundance of one of 60 *Oscillospiraceae* species in our study (HG3A.1161).

Polypharmacy has been associated with various unfavorable health outcomes^7^. Cumulative effects of several medications can be assumed to affect the intestinal environment^45^, and confounding by polypharmacy in gut microbiota studies needs to be considered^5,10,12^. Like in a previous report^12^, polypharmacy associated with differences in the overall gut microbial composition in our study. Further, among those with polypharmacy all three alpha-diversity measures were decreased, which is in line with some^5^ but not all previous studies^12^. Explanations to this could be differences in the study populations and the metagenomic methods. The large population size of our study, high resolution of the metagenomic method together with de novo identification of microbial species allowed us to identify a higher number of species than in most earlier studies, affecting the diversity measurements and making between-studies comparison difficult. Our results yet need to be interpreted with caution, as the number of individuals with polypharmacy in MOS was limited, their medication use differed widely and the differences in diversity measures could also reflect underlying disease states.

In our study, polypharmacy associated with six species of which three, *Enterococcus faecalis*, *Bacteroides uniformis* and one *Eubacterialis* species (HG3A.0137), were not associated with any specific medication use. *E. faecalis,* is a common cause of antibiotic resistant hospital-acquired infection. Our finding is concordant with Nagata et al.^5^ report of a gradual increase of this pathobiont by the number of medications taken. Of the other polypharmacy associated species, *R. mucilaginosa* additionally associated with PPI use*, E. coli* with metformin/antidiabetics and *L. vaginalis* with use of PPIs, ARBs, and calcium-channel blockers*. R. mucilaginosa*, which is a normal habitant of the oral microbiota^46^, is an opportunistic pathogen that has been associated to various diseases. This bacterium has recently been described to harbor a catecholate-siderophore which produces iron-scavenging enterobactin^47^, similar to *E. coli* that gains survival advantage via production of enterobactin^48^. Further, *R. mucilaginosa* may increase virulence of other pathogenic species via a siderophore-sharing mechanism to supply them with iron. Interestingly, synergism between *E. coli* and *B. uniformis* has been suggested, where *E. coli* utilizes d-galactose generated by *B. uniformis* as a source of carbon for its own growth^49^. In the model adjusted for age, sex, and BMI but not for Shannon index, considerably more associations between polypharmacy and species were seen, predominantly negative ones. Taken together, these findings might indicate that polypharmacy suppresses a broad range of species to a certain degree, and thereby paves the way for certain pathobionts to thrive.

Most of the observed associations with GMMs were observed for polypharmacy, with the strongest association noted for dissimilatory nitrate reduction. This path converts nitrate via nitrite to ammonium and represents a common detoxification process in facultative anaerobic bacteria like *E. coli*. Altogether 13 GMMs associated with polypharmacy, and the five species that positively associated with polypharmacy in our study contained between 3 and 11 of the associated GMMs each, with *E. coli* harboring 11 of them. Concordant with our results, a Japanese study^5^ reported positive associations with the number of medications taken and genes related to pyruvate dehydrogenase complex, proline degradation, lactate consumption I and NADH:ferredoxin oxidoreductase and ribose degradation all of which were among GMMs that associated with polypharmacy in our study. Nagata et al., also reported a positive association between polypharmacy usage and fatty acid degradation, which is in line with our findings of higher anaerobic fatty acid beta-oxidation in polypharmacy users.

Our study has several important strengths. To our knowledge, this is the largest study of European general population cohort to report connections between medication use and gut microbiota using high resolution metagenomic sequencing^5,12,24^. Another strength is the high-quality medication data, collected from both questionnaire and medication-register. Further, the data of MOS cohort allows adjustments for known potential confounders such as diet and physical activity. Also, we performed analyses for the medications at different ATC-levels, which enabled identification of associations at both medication-class and substance level. Utilizing these strengths, we replicated several findings of previous studies, but also identified numerous new associations to be validated in future studies.

Our study also has some limitations. First, the study population is Swedish, and although the results align with earlier results in European studies, they may not be generalizable to other populations. Second, the cross-sectional design prohibits any conclusions about causality. Third, we could not address medication dosages, intermittent or continuous use, nor the potential impact of specific co-administrated medications on the observed associations. Fourth, due to the lack of data we could not account for stool consistency as a potential confounder in our analyses. Fifth, adjusting for Shannon index aiming to reduce false positive findings due to the collinearity of the relative abundances of species and Shannon index, might concomitantly have increased bias and decreased power if Shannon index acted as a collider or mediator in some cases. Therefore, we also report all analyses unadjusted for Shannon index. Sixth, adjustments for fiber intake and physical activity could only be performed in the subset of 1475 participants which decreased the power, however, the estimates of most associations remained similar to those in the whole study cohort, indicating that confounding by these factors was limited. Finally, the associations between medication use and the gut microbiota can obviously be confounded by the indications for the treatments, or by other factors that co-vary with the medication.

## Conclusions

Utilizing medication-usage data from complementary sources, we identified many new associations between use of medications, medication-classes, and polypharmacy, and the gut microbiota diversity, species relative abundance and functional potential. Further, we confirmed several previously known associations and demonstrated the association of PPI-associated gut species with their oral counterparts, supporting the view of species transposal from mouth to gut due to these medications. Our study adds new insights to our understanding of the interplay between medications and the gut microbiota composition and function in the general population.

## Supplementary Information

Supplementary Information 1.

Supplementary Tables.

Supplementary Tables.

## Data Availability

The source code and the summary data underlying all figures used to generate the results for the analysis are available upon request from the corresponding author (M.O-M.). Access to pseudonymized microbiota and phenotype data of MOS/MODS requires ethical approval from the Swedish Ethical Review Board and approval from the data access board (https://www.malmo-kohorter.lu.se/uttag).

## Code availability

The source code and the summary data underlying all figures used to generate the results for the analysis are available upon request from the corresponding author (M.O-M.).

## References

[1] Chen, L. *et al.* The long-term genetic stability and individual specificity of the human gut microbiome. *Cell* **184**, 2302–2315. 10.1016/j.cell.2021.03.024 (2021). 33838112 10.1016/j.cell.2021.03.024

[2] Human Microbiome Project, C. Structure, function and diversity of the healthy human microbiome. *Nature* **486**, 207–214. 10.1038/nature11234 (2012). 22699609 10.1038/nature11234 PMC3564958

[3] Falony, G. *et al.* Population-level analysis of gut microbiome variation. *Science* **352**, 560–564. 10.1126/science.aad3503 (2016). 27126039 10.1126/science.aad3503

[4] Zhernakova, A. *et al.* Population-based metagenomics analysis reveals markers for gut microbiome composition and diversity. *Science* **352**, 565–569. 10.1126/science.aad3369 (2016). 27126040 10.1126/science.aad3369 PMC5240844

[5] Nagata, N. *et al.* Population-level metagenomics uncovers distinct effects of multiple medications on the human gut microbiome. *Gastroenterology* **163**, 1038–1052. 10.1053/j.gastro.2022.06.070 (2022). 35788347 10.1053/j.gastro.2022.06.070

[6] Doestzada, M. *et al.* Pharmacomicrobiomics: A novel route towards personalized medicine?. *Protein Cell* **9**, 432–445. 10.1007/s13238-018-0547-2 (2018). 29705929 10.1007/s13238-018-0547-2 PMC5960471

[7] Masnoon, N., Shakib, S., Kalisch-Ellett, L. & Caughey, G. E. What is polypharmacy? A systematic review of definitions. *BMC Geriatr.* **17**, 230. 10.1186/s12877-017-0621-2 (2017). 29017448 10.1186/s12877-017-0621-2 PMC5635569

[8] Gnjidic, D. *et al.* Polypharmacy cutoff and outcomes: Five or more medicines were used to identify community-dwelling older men at risk of different adverse outcomes. *J. Clin. Epidemiol.* **65**, 989–995. 10.1016/j.jclinepi.2012.02.018 (2012). 22742913 10.1016/j.jclinepi.2012.02.018

[9] Wastesson, J. W., Morin, L., Tan, E. C. K. & Johnell, K. An update on the clinical consequences of polypharmacy in older adults: A narrative review. *Expert Opin. Drug Saf.* **17**, 1185–1196. 10.1080/14740338.2018.1546841 (2018). 30540223 10.1080/14740338.2018.1546841

[10] Forslund, S. K. *et al.* Combinatorial, additive and dose-dependent drug-microbiome associations. *Nature* **600**, 500–505. 10.1038/s41586-021-04177-9 (2021). 34880489 10.1038/s41586-021-04177-9

[11] Jackson, M. A. *et al.* Gut microbiota associations with common diseases and prescription medications in a population-based cohort. *Nat. Commun.* **9**, 2655. 10.1038/s41467-018-05184-7 (2018). 29985401 10.1038/s41467-018-05184-7 PMC6037668

[12] Vich Vila, A. *et al.* Impact of commonly used drugs on the composition and metabolic function of the gut microbiota. *Nat. Commun.* **11**, 362. 10.1038/s41467-019-14177-z (2020). 31953381 10.1038/s41467-019-14177-z PMC6969170

[13] Forslund, K. *et al.* Disentangling type 2 diabetes and metformin treatment signatures in the human gut microbiota. *Nature* **528**, 262–266. 10.1038/nature15766 (2015). 26633628 10.1038/nature15766 PMC4681099

[14] Wu, H. *et al.* Metformin alters the gut microbiome of individuals with treatment-naive type 2 diabetes, contributing to the therapeutic effects of the drug. *Nat. Med.* **23**, 850–858. 10.1038/nm.4345 (2017). 28530702 10.1038/nm.4345

[15] de la Cuesta-Zuluaga, J. *et al.* Metformin is associated with higher relative abundance of mucin-degrading *Akkermansia muciniphila* and several short-chain fatty acid-producing microbiota in the gut. *Diabetes Care* **40**, 54–62. 10.2337/dc16-1324 (2017). 27999002 10.2337/dc16-1324

[16] Freedberg, D. E. *et al.* Proton pump inhibitors alter specific taxa in the human gastrointestinal microbiome: A crossover trial. *Gastroenterology* **149**, 883–885. 10.1053/j.gastro.2015.06.043 (2015). 26164495 10.1053/j.gastro.2015.06.043 PMC4584196

[17] Imhann, F. *et al.* The influence of proton pump inhibitors and other commonly used medication on the gut microbiota. *Gut Microbes* **8**, 351–358. 10.1080/19490976.2017.1284732 (2017). 28118083 10.1080/19490976.2017.1284732 PMC5570416

[18] Brunkwall, L. *et al.* The Malmo Offspring Study (MOS): Design, methods and first results. *Eur. J. Epidemiol.* **36**, 103–116. 10.1007/s10654-020-00695-4 (2021). 33222051 10.1007/s10654-020-00695-4 PMC7847466

[19] Nybacka, S. *et al.* Comparison of a web-based food record tool and a food-frequency questionnaire and objective validation using the doubly labelled water technique in a Swedish middle-aged population. *J. Nutr. Sci.* **5**, e39. 10.1017/jns.2016.29 (2016). 27752306 10.1017/jns.2016.29 PMC5048186

[20] Dekkers, K. F. *et al.* An online atlas of human plasma metabolite signatures of gut microbiome composition. *Nat. Commun.* **13**, 5370. 10.1038/s41467-022-33050-0 (2022). 36151114 10.1038/s41467-022-33050-0 PMC9508139

[21] Nielsen, H. B. *et al.* Identification and assembly of genomes and genetic elements in complex metagenomic samples without using reference genomes. *Nat. Biotechnol.* **32**, 822–828. 10.1038/nbt.2939 (2014). 24997787 10.1038/nbt.2939

[22] Vieira-Silva, S. *et al.* Species-function relationships shape ecological properties of the human gut microbiome. *Nat. Microbiol.* **1**, 16088. 10.1038/nmicrobiol.2016.88 (2016). 27573110 10.1038/nmicrobiol.2016.88

[23] Dixon, P. VEGAN a package of R functions for community ecology. *J. Veg. Sci.* **2003**, 927–930 (2003).

[24] Aasmets, O., Krigul, K. L., Lull, K., Metspalu, A. & Org, E. Gut metagenome associations with extensive digital health data in a volunteer-based Estonian microbiome cohort. *Nat. Commun.* **13**, 869. 10.1038/s41467-022-28464-9 (2022). 35169130 10.1038/s41467-022-28464-9 PMC8847343

[25] Lloyd-Price, J., Abu-Ali, G. & Huttenhower, C. The healthy human microbiome. *Genome Med.* **8**, 51. 10.1186/s13073-016-0307-y (2016). 27122046 10.1186/s13073-016-0307-y PMC4848870

[26] Jackson, M. A. *et al.* Proton pump inhibitors alter the composition of the gut microbiota. *Gut* **65**, 749–756. 10.1136/gutjnl-2015-310861 (2016). 26719299 10.1136/gutjnl-2015-310861 PMC4853574

[27] Weersma, R. K., Zhernakova, A. & Fu, J. Interaction between drugs and the gut microbiome. *Gut* **69**, 1510–1519. 10.1136/gutjnl-2019-320204 (2020). 32409589 10.1136/gutjnl-2019-320204 PMC7398478

[28] Imhann, F. *et al.* Proton pump inhibitors affect the gut microbiome. *Gut* **65**, 740–748. 10.1136/gutjnl-2015-310376 (2016). 26657899 10.1136/gutjnl-2015-310376 PMC4853569

[29] Mueller, N. T. *et al.* Metformin affects gut microbiome composition and function and circulating short-chain fatty acids: A randomized trial. *Diabetes Care* **44**, 1462–1471. 10.2337/dc20-2257 (2021). 34006565 10.2337/dc20-2257 PMC8323185

[30] Sivertsen, A. *et al.**Escherichia marmotae*—a human pathogen easily misidentified as *Escherichia coli*. *Microbiol. Spectr.* **10**, 56. 10.1128/spectrum.02035-21 (2022). 10.1128/spectrum.02035-21 PMC9045135 35380461

[31] Ricaboni, D., Mailhe, M., Khelaifia, S., Raoult, D. & Million, M. *Romboutsia timonensis*, a new species isolated from human gut. *New Microbes New Infect.* **12**, 6–7. 10.1016/j.nmni.2016.04.001 (2016). 27200178 10.1016/j.nmni.2016.04.001 PMC4864248

[32] Collins, S. M., Surette, M. & Bercik, P. The interplay between the intestinal microbiota and the brain. *Nat. Rev. Microbiol.* **10**, 735–742. 10.1038/nrmicro2876 (2012). 23000955 10.1038/nrmicro2876

[33] Valles-Colomer, M. *et al.* The neuroactive potential of the human gut microbiota in quality of life and depression. *Nat. Microbiol.* **4**, 623–632. 10.1038/s41564-018-0337-x (2019). 30718848 10.1038/s41564-018-0337-x

[34] Zheng, P. *et al.* Gut microbiome remodeling induces depressive-like behaviors through a pathway mediated by the host’s metabolism. *Mol. Psychiatry* **21**, 786–796. 10.1038/mp.2016.44 (2016). 27067014 10.1038/mp.2016.44

[35] Rukavishnikov, G. *et al.* Antimicrobial activity of antidepressants on normal gut microbiota: Results of the in vitro study. *Front. Behav. Neurosci.* **17**, 1132127. 10.3389/fnbeh.2023.1132127 (2023). 37035624 10.3389/fnbeh.2023.1132127 PMC10073483

[36] Maseda, D. & Ricciotti, E. NSAID-gut microbiota interactions. *Front. Pharmacol.* **11**, 1153. 10.3389/fphar.2020.01153 (2020). 32848762 10.3389/fphar.2020.01153 PMC7426480

[37] Szostak, N. *et al.* Host factors associated with gut mycobiome structure. *mSystems* **8**, e0098622. 10.1128/msystems.00986-22 (2023). 36786595 10.1128/msystems.00986-22 PMC10134842

[38] Jiang, T. T. *et al.* Commensal fungi recapitulate the protective benefits of intestinal bacteria. *Cell Host Microbe* **22**, 809–816. 10.1016/j.chom.2017.10.013 (2017). 29174402 10.1016/j.chom.2017.10.013 PMC5730478

[39] Walker, L. J. *et al.* Anti-Saccharomyces cerevisiae antibodies (ASCA) in Crohn’s disease are associated with disease severity but not NOD2/CARD15 mutations. *Clin. Exp. Immunol.* **135**, 490–496. 10.1111/j.1365-2249.2003.02392.x (2004). 15008984 10.1111/j.1365-2249.2003.02392.x PMC1808965

[40] Rogers, M. A. M. & Aronoff, D. M. The influence of non-steroidal anti-inflammatory drugs on the gut microbiome. *Clin. Microbiol. Infect.* **22**, 178. 10.1016/j.cmi.2015.10.003 (2016). 10.1016/j.cmi.2015.10.003 PMC4754147 26482265

[41] Wu, D. *et al.* Candesartan attenuates hypertension-associated pathophysiological alterations in the gut. *Biomed. Pharmacother.* **116**, 109040. 10.1016/j.biopha.2019.109040 (2019). 31170664 10.1016/j.biopha.2019.109040

[42] Robles-Vera, I. *et al.* Changes to the gut microbiota induced by losartan contributes to its antihypertensive effects. *Br. J. Pharmacol.* **177**, 2006–2023. 10.1111/bph.14965 (2020). 31883108 10.1111/bph.14965 PMC7161554

[43] Li, Y. *et al.* Amlodipine, an anti-hypertensive drug, alleviates non-alcoholic fatty liver disease by modulating gut microbiota. *Br. J. Pharmacol.* **179**, 2054–2077. 10.1111/bph.15768 (2022). 34862599 10.1111/bph.15768

[44] Verhaar, B. J. H., Prodan, A., Nieuwdorp, M. & Muller, M. Gut microbiota in hypertension and atherosclerosis: A review. *Nutrients* **2020**, 12. 10.3390/nu12102982 (2020). 10.3390/nu12102982 PMC7601560 33003455

[45] Maier, L. *et al.* Extensive impact of non-antibiotic drugs on human gut bacteria. *Nature* **555**, 623–628. 10.1038/nature25979 (2018). 29555994 10.1038/nature25979 PMC6108420

[46] Maraki, S. & Papadakis, I. S. *Rothia mucilaginosa* pneumonia: A literature review. *Infect. Dis. (Lond.)* **47**, 125–129. 10.3109/00365548.2014.980843 (2015). 25664502 10.3109/00365548.2014.980843

[47] Uranga, C. C., Arroyo, P. Jr., Duggan, B. M., Gerwick, W. H. & Edlund, A. Commensal oral rothia mucilaginosa produces enterobactin, a metal-chelating siderophore. *mSystems* **2020**, 5. 10.1128/mSystems.00161-20 (2020). 10.1128/mSystems.00161-20 PMC7190385 32345739

[48] Singh, V. *et al.* Interplay between enterobactin, myeloperoxidase and lipocalin 2 regulates *E. coli* survival in the inflamed gut. *Nat. Commun.* **6**, 7113. 10.1038/ncomms8113 (2015). 25964185 10.1038/ncomms8113 PMC6336494

[49] Li, M. *et al.* Isolation and characterization of an agaro-oligosaccharide (AO)-hydrolyzing bacterium from the gut microflora of Chinese individuals. *PLoS One* **9**, e91106. 10.1371/journal.pone.0091106 (2014). 24622338 10.1371/journal.pone.0091106 PMC3951304

[50] Letunic, I. & Bork, P. Interactive Tree of Life (iTOL) v6: Recent updates to the phylogenetic tree display and annotation tool. *Nucleic Acids Res.*10.1093/nar/gkae268 (2024). 38613393 10.1093/nar/gkae268 PMC11223838

