# Supplementary material for: New connections of medication use and polypharmacy with the gut microbiota composition and functional potential in a large population
Source: Sci Rep. 2024 Oct 10;14:23723. doi: 10.1038/s41598-024-71571-4 (PMC11467196; doi:10.1038/s41598-024-71571-4)
Supplement: Supplementary file 1 — Supplementary Information 1. [file 41598_2024_71571_MOESM1_ESM.pdf]

Supplementary information

Novel connections of medication use and polypharmacy with the gut microbiota composition and functional potential in a large population

Anna Larsson, MD[1], Ulrika Ericson, PhD[1], Daniel Jönsson, DDS, PhD[1,2,3], Mariam Miari, MSc[1], Paschalis Athanasiadis, MSc[1], Gabriel Baldanzi, MD[4], Louise Brunkwall, PhD[1], Sophie Hellstrand, PhD[1], Björn Klinge, DDS, PhD[3,5], Olle Melander, PhD, MD[1], Peter M Nilsson, PhD, MD[1], Tove Fall, PhD[4,6], Marlena Maziarz, PhD[1] and Marju Orho-Melander, PhD[1*]

Supplementary results

Additional adjustments for fiber intake and leisure time physical activity level

The reduction of the sample size from 2223 to 1475, due to lack of data for fiber intake and physical activity, resulted in that 22 of the 97 medication-species associations, and three of seven medication-GMM associations, remained statistically significant at $q<0.05$. However, all except one of these associations remained nominally significant ($p<0.05$) in the reduced study sample and the association results were similar in the models with and without adjustments for fiber intake and physical activity (Supplementary Tables 4, 5, 30, 37, 38, 49). Likewise, of the original six associations between species and polypharmacy, two remained at $q<0.05$ after reducing the sample size (n=1475) (Supplementary Tables 19, 20 and 31), four remained nominally significant and the associations were similar in the models with and without adjustments for fiber intake and physical activity. Regarding GMM associations with polypharmacy, none of these remained significant ($q>0.05$) in the reduced sample size (Supplementary Tables 45, 46, 50). Further, four of the 18 correlations between medications and Shannon index remained statistically significant ($q<0.05$) in the reduced sample, and the associations were largely unaffected by the additional adjustments (Supplementary Tables 11, 12 and 32). The correlation between polypharmacy and Shannon index remained in the subset of 1475 individuals and was not affected by the additional adjustments (Supplementary Tables 25, 26 and 33). The correlation-coefficients (*rho*) for the vast majority of all the original associations in the subset of 1475 participants only changed marginally after the additional adjustments (Supplementary Tables 30-33).

Supplementary information

Sensitivity analyses

We performed three sensitivity analyses excluding participants with 1) antibiotic usage the last six months; 2) antibiotic usage the last 12 months; and 3) polypharmacy. Altogether 13 and 28 of the 97 medication-species associations, and none and four of the medication-GMM associations from the main model remained ($q<0.05$), in sensitivity analyses 1 and 2 (Supplementary Tables 6, 7 and 30, 39, 40, 49), respectively. In sensitivity analysis excluding participants with polypharmacy, 32 medication-species associations but none of the GMM-associations remained (Supplementary Tables 8 and 30, 41, 49). For most of the 97 original associations, no major changes were seen in correlation coefficients in the sensitivity analyses. In all three sensitivity analyses, the greatest decrease in the correlation coefficients (>20%) were observed for associations with medications or medication classes belonging to antidiabetic medicines, medications for acid related disorders, ARBs, nasal preparations or combined mucolytic cough mixtures. Eight associations between medication-variables and species were robust ($q<0.05$) in all three sensitivity analyses and those belonged to medications for acid related disorders, antidepressants, and selective betablockers (Supplementary Table 30).

The original six correlations between polypharmacy and gut species did not remain ($q<0.05$) in the two sensitivity analyses excluding antibiotic users (Supplementary Tables 21, 22 and 31). No major changes were seen in the strength of correlations between polypharmacy and *E. coli*, *Eubacteriales* sp. (HG3A.0137) and *B. uniformis*. Concerning sensitivity analyses of GMM associations, the correlation coefficients generally showed negligible differences compared to those of main analyses, however with more noticeable variations for the associations between GMMs and polypharmacy, which may be explained by the limited number of participants with polypharmacy in these analyses (Supplementary Tables 39, 40, 41, 47, 48, 49, 50).

Supplementary information

Concerning the correlations between Shannon index and medications, five of the 18 original associations remained ($q<0.05$) in sensitivity analysis 1, but not in sensitivity analysis 2 and 3 (Supplementary Tables 13, 14, 15 and 32). Most correlation coefficients remained similar. In the case of paracetamol and metformin, notable reductions in correlation coefficients were seen when excluding polypharmacy users (sensitivity analysis 3) and this also applied to corticosteroids and levothyroxine when excluding antibiotic users in sensitivity analysis 1 and in all three sensitivity analyses in the case of `Drugs for acid related disorders´, including PPIs. Excluding those with previous antibiotic usage did not seem to affect the association between polypharmacy and Shannon index ($p<0.067$ and $p<0.05$ in sensitivity analyses 1 and 2), (Supplementary Tables 27, 28 and 33).

Supplementary information

Supplementary Figures

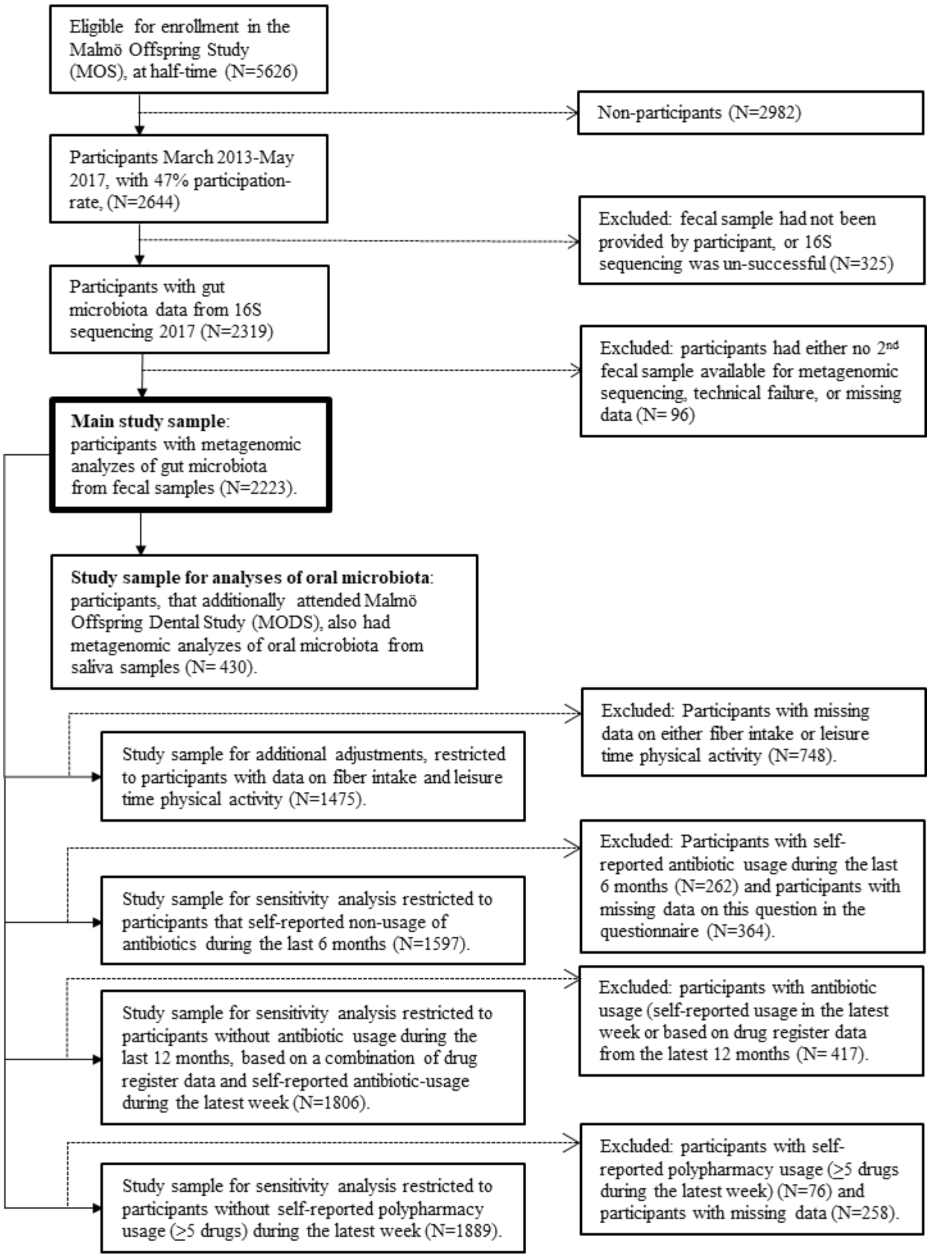

Supplementary Figure 1. Flowchart depicting the cross-sectional study design, with 2223 participants in the main study sample.

Supplementary information

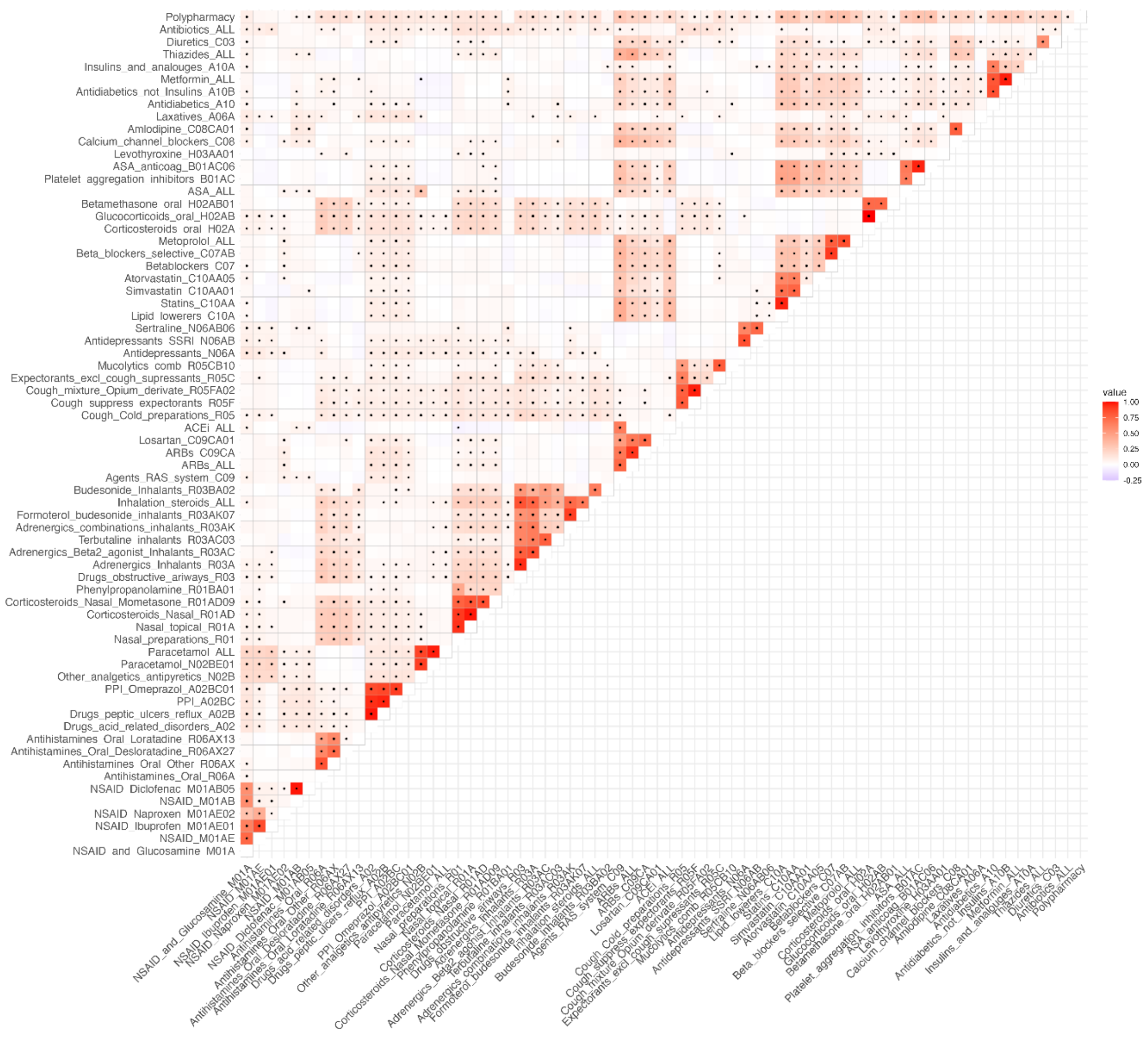

Supplementary Figure 2. Co-occurrence of medication usage. The heatmap presents all pairwise correlations between the 67 medication variables and polypharmacy. Red indicates positive and blue negative correlations, ● represent significance at $p<0.05$. Please note that the largest negative correlation coefficient is -0.045, hence so little blue is visible in the plot.

Supplementary information

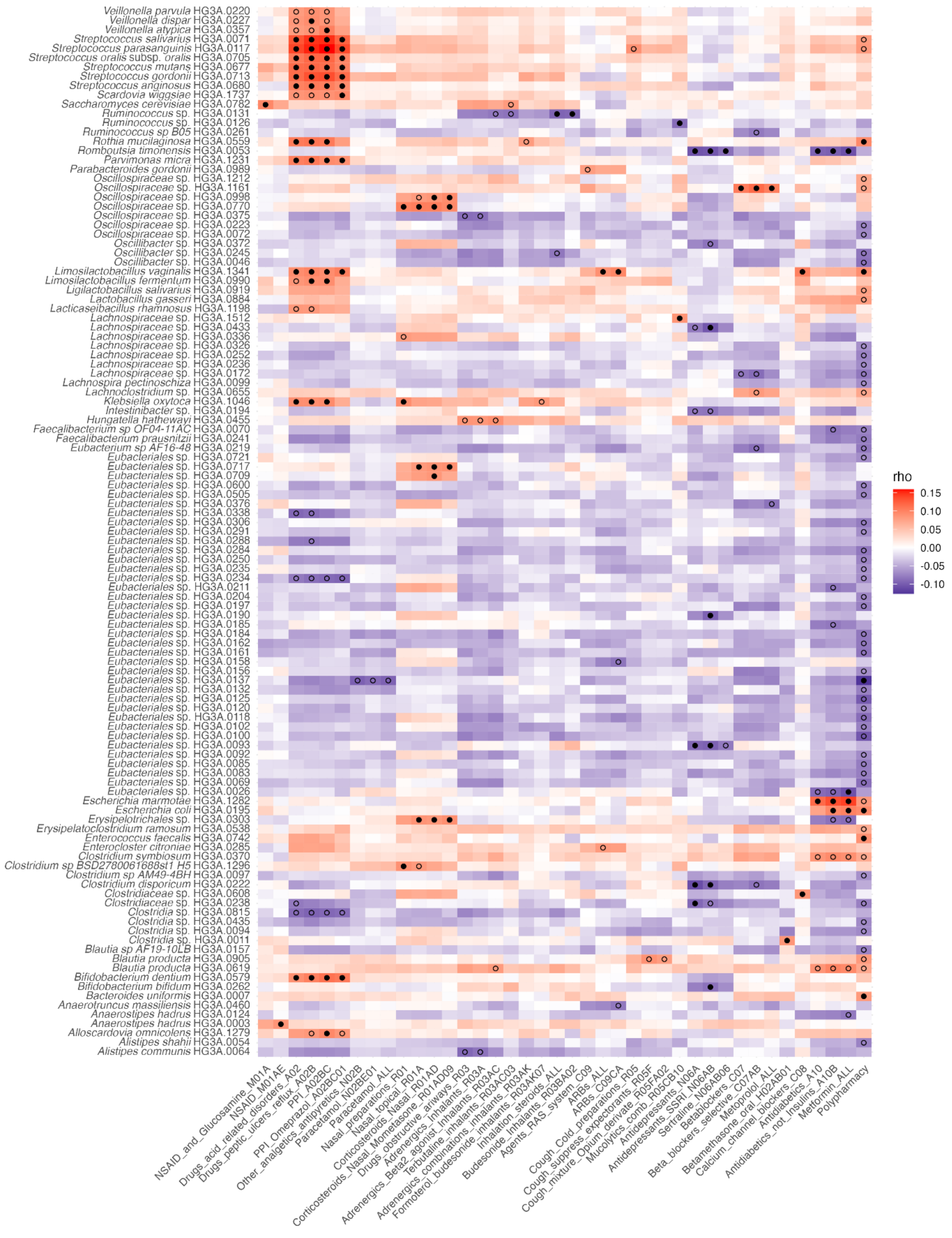

Supplementary Figure 3. Associations between gut species and medication use (Model 2). The heat map displays partial Spearman’s rank correlations, for all medications/medication classes and polypharmacy (columns) that were significantly associated with any of the gut species (rows), adjusted for age, sex, and BMI. Red indicates positive, and blue indicates negative correlations. Correlations that were statistically significant (FDR adjusted q-value <0.05) in Model 2 only are indicated by ○, correlations that were statistically significant in both models 2 and 3 (main model, displayed in Fig. 3) are indicated by ●. No correlations were significant only in model 3.

Supplementary information

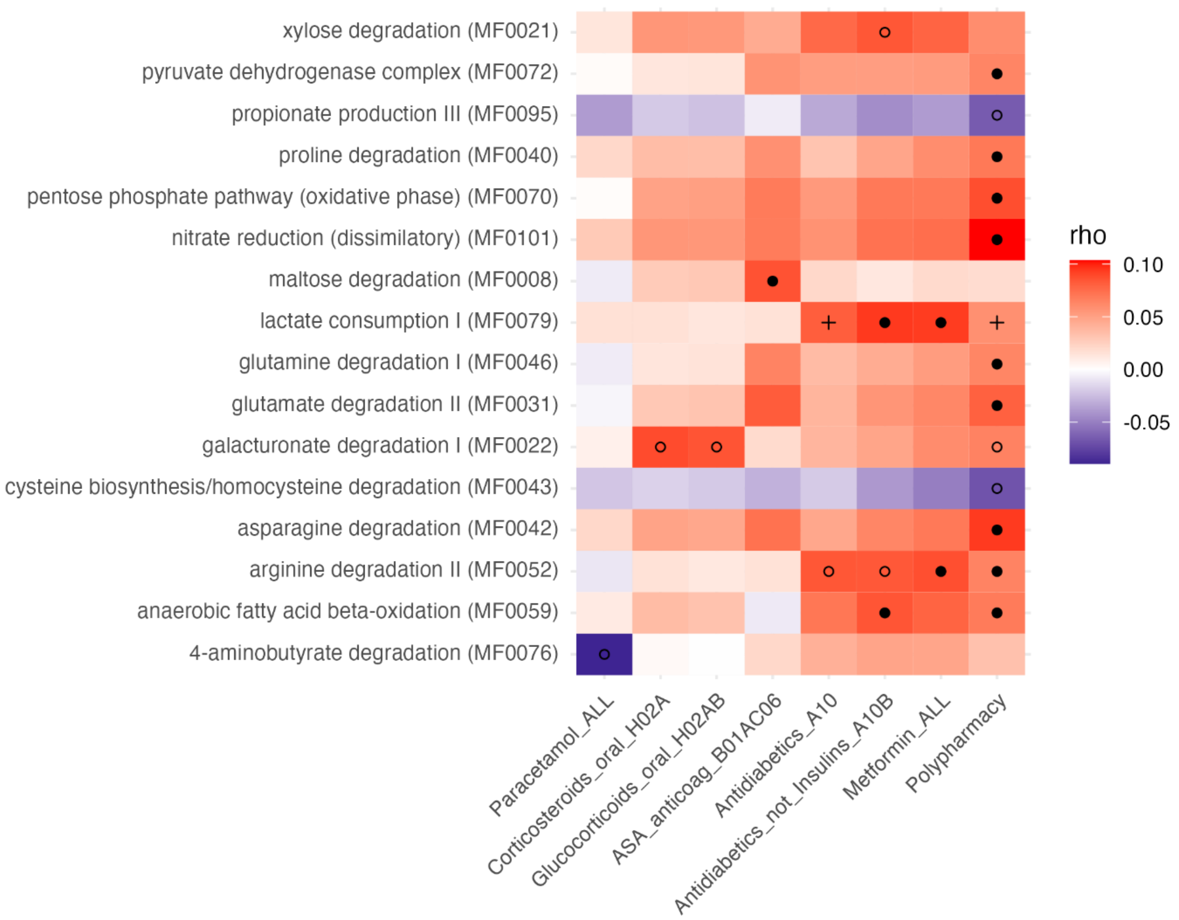

Supplementary Figure 4. Associations between gut metabolic modules and medication use (Model 2). The heat map displays partial Spearman's rank correlations, for all medications/medication classes and polypharmacy (columns) that were significantly associated with any of the gut metabolic modules (rows), adjusted for age, sex, and BMI. Red indicates positive correlations, and blue indicates negative correlations. Correlations that were statistically significant (FDR adjusted q-value <0.05) in Model 2 only are indicated by ○, correlations that were significant in both models 2 and 3 (main model, displayed in Fig. 4) are indicated by ●. Correlations that were significant only in model 3 are indicated by +.
